# Supplementary material for: Genome Sequence of the Bacteriophage CL31 and Interaction with the Host Strain Corynebacterium glutamicum ATCC 13032
Source: Viruses. 2021 Mar 17;13(3):495. doi: 10.3390/v13030495 (PMC8002715; doi:10.3390/v13030495)
Supplement: Supplementary file 1 [file viruses-13-00495-s001.zip › 20210310_CL31_Hünnefeld_Viruses_SI.pdf]

viruses

MDPI

Supplementary Information

Genome sequence of the bacteriophage CL31 and analysis of its characteristics and host interactions

Max Hünnefeld, Ulrike Viets, Vikas Sharma, Astrid Wirtz, Aël Hardy, and Julia Frunzke*

Institute of Bio- and Geosciences: IBG-1, Forschungszentrum Jülich, 52425 Jülich, Germany; m.huennefeld@fz-juelich.de (M.H.), u.viets@fz-juelich.de (U.V.), v.sharma@fz-juelich.de (V.S.), a.wirtz@fz-juelich.de (A.W.), a.hardy@fz-juelich.de (A.H.)

* Correspondence: j.frunzke@fz-juelich.de, Tel.: +49-2461-615430

List of all supplementary files

Table S1: Oligonucleotides used in this study.
Table S2: Construction of plasmids used in this study.
Table S3 (excel): Gene prediction and annotation of the CL31 genome.
Table S4 (excel): Distances of CL31 in comparison with other phage genomes.
Table S5 (excel): Transcriptome analysis of host cells and CL31 during infection of MB001 cells.
Table S6: Lifestyle prediction and validation of CL31 and other corynephages.

Figure S1: PCR and Sanger sequencing of singles plaques to close the CL31 genome.
Figure S2: Synteny plot comparing the corynephages CL31 and Φ16.
Figure S3: Spot assays of the infection dynamics analysis of CL31 with different hosts.
Figure S4: Infection curve for RNA-seq analysis.

Table S1. Oligonucleotides used in this study.

Number	Sequence (5′-3′)
1	CCTGCAGGTCGACTCTAGAGAGCATGGAATCGCCACCGAT
2	CATTCCACTATAGAAACTGTTTTTTAAAGGAGAACCATGTCTGA
3	TAAAAAACAGTTTCTATAGTGGAATGTTACCGTGCTTTTTAGC
4	CCCAGTTCGCTGACTGCTTGGATAT
5	CAAGCAGTCAGCGAACTGGGTTGGC
6	CTAAGAAACCATGACTGCAGCACAGACCA
7	CTGCAGTCATGGTTTCTTAGTCTAAATCCTGAAGG
8	TTGTAAAACGACGGCCAGTGAGACCACATGCTCAACCCGT
9	CCTGCAGGTCGACTCTAGAGGAGGTCTCGCACAGCCTTGGT
10	GCCGACAAGGACGCCCGCTTCATCC
11	AAGCGGGCGTCCTTGTCGGCGGCGT
12	TTGTAAAACGACGGCCAGTGACCTGTGACCACTGACTTTGTTATCGGCGT
13	CCTGCAGGTCGACTCTAGAGAATCTGAGGGCTGCGGCC
14	GGAAGAATTAGTAACGGAGAGCTGACGGAAG
15	CTCTCCGTTACTAATTCTTCCGAGAAATCTCATCAATGC
16	AGTGGTCCACGACGCCGATGACATG
17	CATCGGCGTCGTGGACCACTGCTCC
18	AGGCCTCAATATGGAACAGAGCCAAT
19	TCTGTTCCATATTGAGGCCTCAGCCT
20	TTGTAAAACGACGGCCAGTGCGCTCCGCCGTTGGC
21	GTGTCCAATTCCAGATCCACATCGT
22	ACGATGTGGATCTGGAATTGGACAC
23	CTGGTTGGTGCCAGGCAGGAAG
24	GGTGGTCACGTGTACTCCCCTG
25	CTGCAGCCTTGGTGTCCTGAGT
26	CACACAAGAACCCTGCACAACGC
27	GGTCATGCATGTCGTAGCCCTGGTTA
28	TAACCAGGGCTACGACATGCATGACC
29	CCACGACGAAGATGATCGGGAT
30	ATGTATGTCACCGGCCCAGCC
31	CCCGACAGAACTGCGAAGATGG
32	GGAGCTCATTTGAGTCGCCGC
33	GCATGTTCTTCTTCTCTGGTAGCGG
34	CGCGATACCATGCTCATTGCCAG
35	GTCGCTGACGTGGTAATCATGGTGG
36	CGGATCCTTTGTGGAGATCGGCG
37	CCGCTACCAGAGAAGAAGAACATGC
38	CTGGCAATGAGCATGGTATCGCG
39	CCACCATGATTACCACGTCAGCGAC
40	CGCCGATCTCCACAAAGGATCCG
41	GATTTCAATTCCGGAACGCTCGGTGAG
42	GGCTACGACGGATTCAACACCGAT
43	ATGCCAGCGATTTCGCCAGC
44	CGAGGAAGCCGTAAAGCGCC
45	GCGTCAACATATGCACGCTGCA
46	GCCAACATGGATCCTCAGCAGC
47	GCGCAGATCCGCTAGCTTCT
48	CCTCGTTTTGGTTGAGGCGG
49	GCTTTCGTACTGAGATCCGCGATCC
50	CGCAGGTGGGGGAGGATTCTTGAA

Table S2: Construction of plasmids used in this study. Numbers represent oligonucleotide pairs used for PCR (see Table A1). The restriction enzymes were used for linearization of the vectors and plasmids were assembled using Gibson assembly (multiple primer pairs indicate that constructs were assembled using multiple fragments). To verify the plasmids and the integrations using sequencing, the following primers were used: *accD2:* 21 – 26; *accD3*: 27 -32; *pks*: 33 – 48.

Plasmid	Template	Primers	Vector	Restriction Enzymes
pK19*mobsacB*-*accD2*_G1049A	*C. glutamicum* chromosome	1 + 2, 3 + 4, 5 + 6, 7 + 8	pK19*mobsacB*	*BamHI *EcoRI
pK19*mobsacB*-*accD3*_G1022A	*C. glutamicum* chromosome	9 + 10, 11 + 12	pK19*mobsacB*	*BamHI *EcoRI
pK19*mobsacB*-*pks*_T2656G	*C. glutamicum* chromosome	13 + 14, 15 + 16, 17 + 18, 19 + 20	pK19*mobsacB*	*BamHI *EcoRI

Table S6. Prediction and (if available) experimental proof of the life style of different corynephages. Please note, we did not identify an integrase gene in the genome of CL31. Therefore, the life style predictions have to be treated with care and further experiments are required.

Phage	Domains (temperate)	Domains (virulent)	Predicted Life Style	Experimental	Reference
CL31	5	1	Temperate	-	This work
Phi16	7	3	Temperate	Temperate	[1,2]
Phi673	0	3	Virulent	Virulent	[3]
Phi674	0	6	Virulent	Virulent	[3]

Figure S1

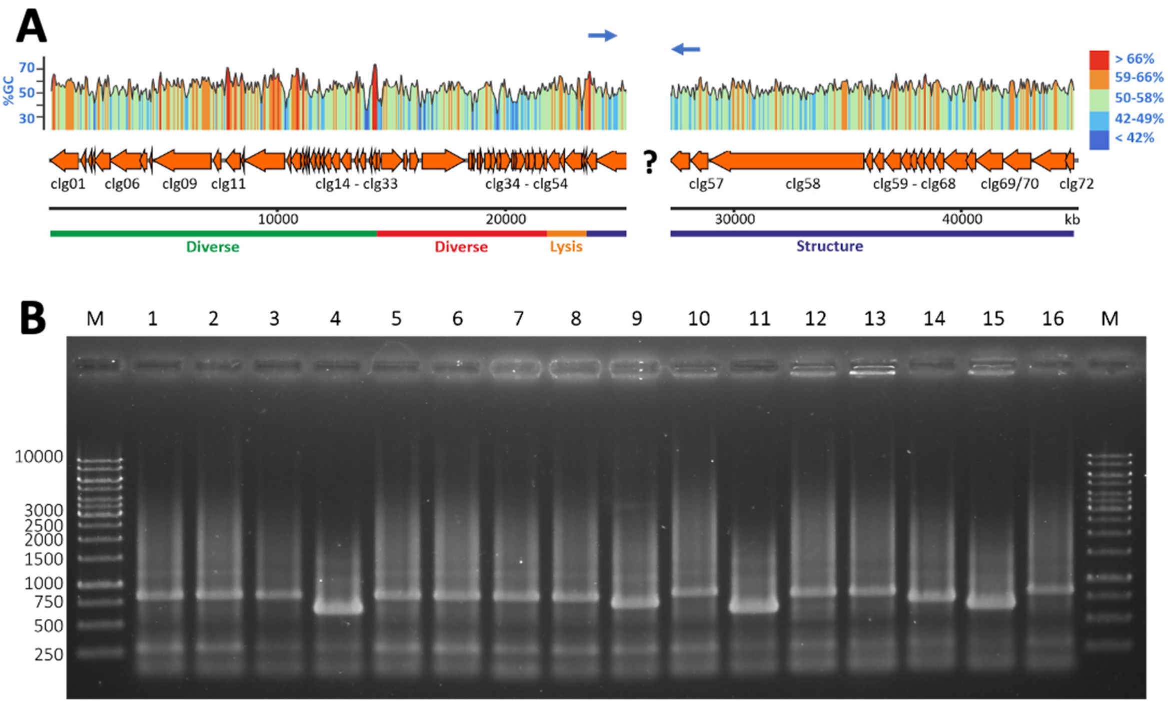

Figure S1: PCR and Sanger sequencing of singles plaques to close the CL31 genome. (A) Schematic representation of the position of a repeat region in the CL31 genome. This region could not be correctly assembled based on Illumina reads and thus was further analyzed with Sanger sequencing. The blue arrows indicate the approximate position of the primers used for PCR amplification and sequencing. (B) Primers 49 and 50 were used to amplify the area shown in A from single CL31 plaques (exemplary plate shown in Figure 2A of the main text). Three different product sizes were obtained. For sequencing three different sizes were chosen (Lane 1, 4, 14).

Figure S2

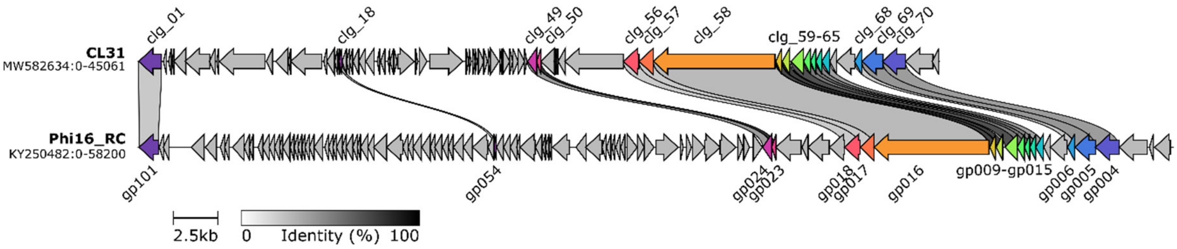

Figure S2: Synteny plot comparing the corynephages CL31 and Φ16. The genomes of the corynephages CL31 and Phi16 (as reverse complement) were compared with regard to identity of single genes. For this purpose, the tool Clinker was used with standard parameters [4].

Figure S3

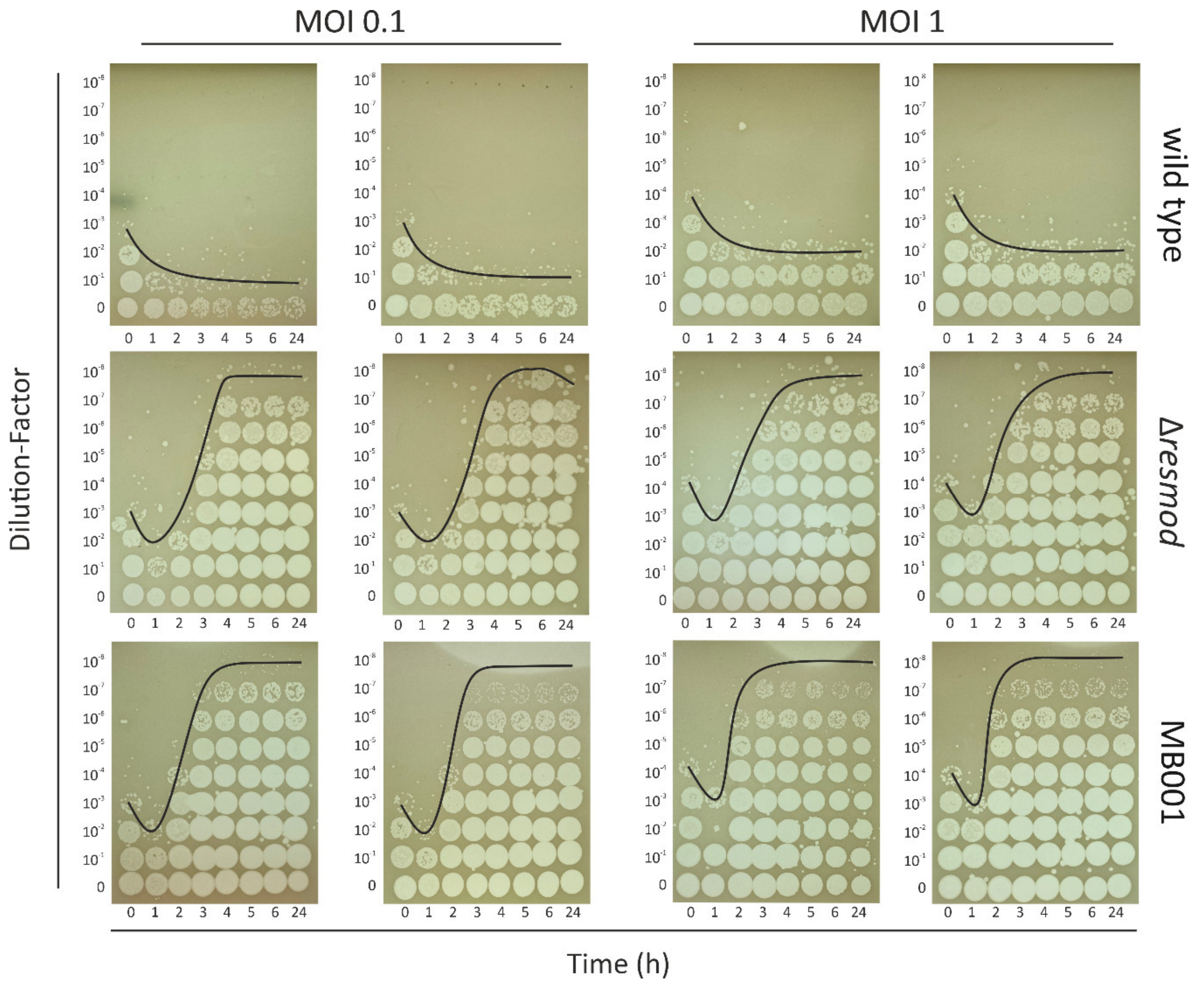

Figure S3: Plate assays as infection dynamics analysis. Infection assay of CL31 with different host strains was used to determine the phage titer at different time points of incubation. For this purpose, bacterial cells were incubated with CL31 in BHI medium with an initial MOI of 0.1 or 1. At the described time points, samples were extracted and 3 µl of the centrifuged supernatant were spotted on a MB001 lawn (OD_{600} 0.5).

Figure S4

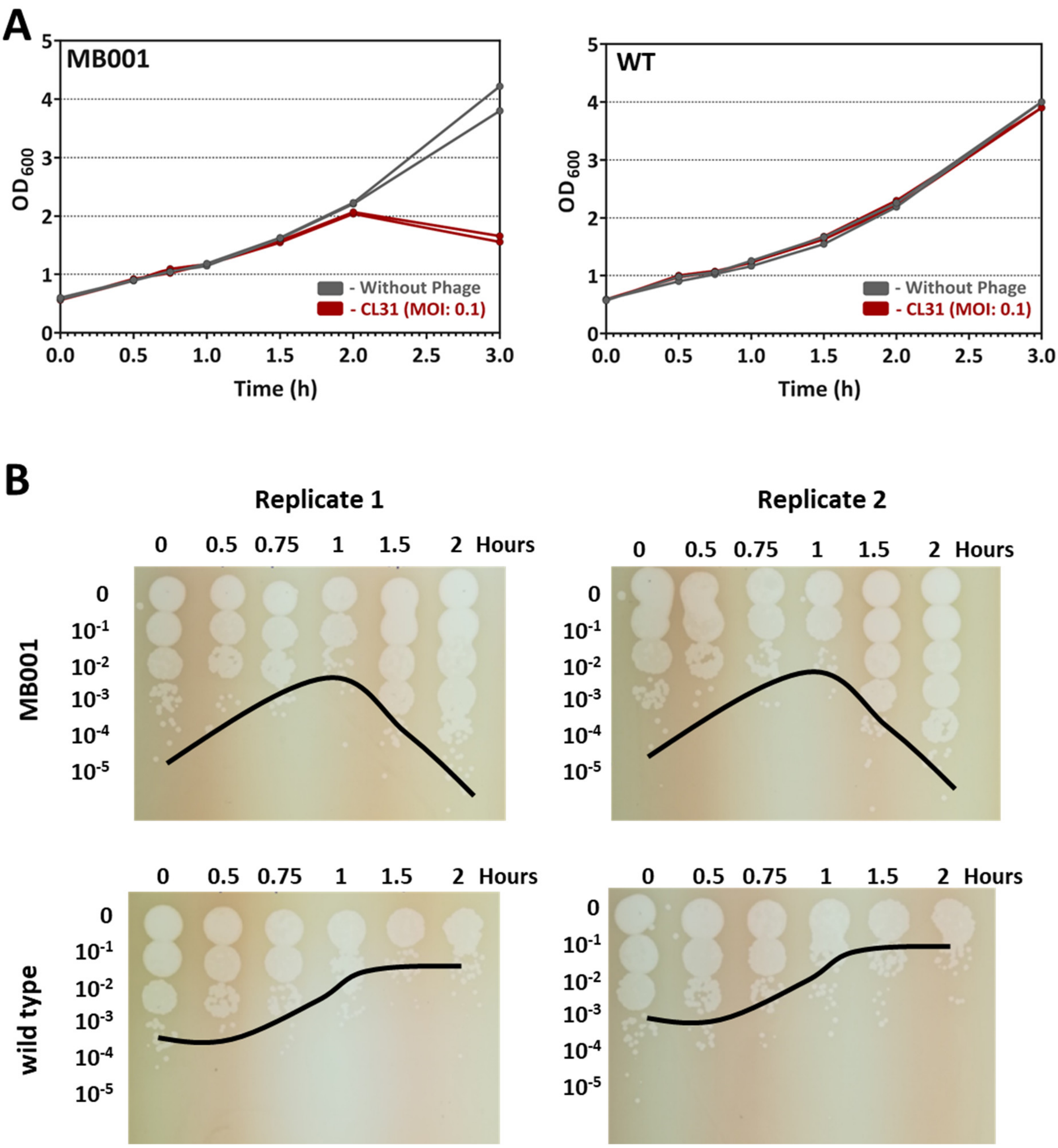

Figure S4: Infection curve for RNA-seq analysis. To determine the best point for RNA-seq analysis, an infection assay of CL31 with wild type or MB001 strains was used to determine the phage titer as well as the growth state (OD_{600} measurements) during incubation. For this purpose, bacterial cells (Starting-OD_{600} 0.5) were incubated with CL31 in BHI medium with an initial MOI of 0.1. At the described time points, samples were extracted, OD measurements were conducted, and 3 µl of the centrifuged supernatant were spotted on a MB001 lawn (OD_{600} 0.5). The resulting plates were analysed after an overnight incubation. To cover the moment of highest intracellular phage activity, 1h after cultivation start was chosen to be used for RNA preparation. This represents the point before phage titer increase.

References

1. Moreau, S.; Leret, V.; Le Marrec, C.; Varangot, H.; Ayache, m.; Bonnassie, S.; Blanco, C.; Trautwetter, A. Prophage distribution in coryneform bacteria. *Res. Microbiol.* **1995**, *146*, 493–505, doi:10.1016/0923-2508(96)80295-6.
2. Lobanova, J.S.; Gak, E.R.; Andreeva, I.G.; Rybak, K. V.; Krylov, A.A.; Mashko, S. V. Complete nucleotide sequence and annotation of the temperate corynephage ϕ16 genome. *Arch. Virol.* **2017**, *162*, 2489–2492, doi:10.1007/s00705-017-3383-4.
3. Yomantas, Y.A.V. V.; Abalakina, E.G.; Lobanova, J.S.; Mamontov, V.A.; Stoynova, N. V.; Mashko, S. V. Complete nucleotide sequences and annotations of φ673 and φ674, two newly characterised lytic phages of Corynebacterium glutamicum ATCC 13032. *Arch. Virol.* **2018**, *163*, 1–4, doi:10.1007/s00705-018-3867-x.
4. Gilchrist, C.L.M.; Chooi, Y.-H. Clinker & Clustermap.Js: Automatic Generation of Gene Cluster Comparison Figures. *Bioinformatics* **2021**, doi:10.1093/bioinformatics/btab007.
